# Synthetic Data for Sharing and Exploration in High-Performance Sport: Considerations for Application

**DOI:** 10.1007/s40279-025-02221-6

**Published:** 2025-06-26

**Authors:** John Warmenhoven, Franco M. Impellizzeri, Ian Shrier, Andrew D. Vigotsky, Lorenzo Lolli, Paolo Menaspà, Aaron J. Coutts, Maurizio Fanchini, Giles Hooker

**Affiliations:** 1 School of Sport, Exercise and Rehabilitation and Human Performance Research Centre, University of Technology Sydney (UTS), Sydney, Australia; 2 Centre for Clinical Epidemiology, Lady Davis Institute, Jewish General Hospital, McGill University, Montreal, Canada; 3 Departments of Biomedical Engineering and Statistics, Northwestern University, Evanston, IL USA; 4 Department of Neuroscience, Northwestern University, Chicago, IL USA; 5 Department of Sport and Exercise Sciences, Institute of Sport, Manchester Metropolitan University, Manchester, UK; 6 Australian Institute of Sport, Australian Sports Commission, Canberra, Australia; 7 AS Roma Performance Department, AS Roma Football Club, Rome, Italy; 8 Department of Neurosciences, Biomedicine and Movement Sciences, University of Verona, Verona, Italy; 9 Research School of Finance, Actuarial Science and Statistics, Australian National University, Canberra, Australia; 10 The Wharton School, University of Pennsylvania, Philadelphia, USA

## Abstract

**Background:**

Synthetic data represent alternative data sources generated using mathematical procedures to address specific issues in research and practice. Synthetic data have emerging applications in clinical and medical data contexts and may assist in overcoming privacy issues to help support open science practice.

**Objective:**

The present study discusses the applicability of an established synthetic data generation process using sequential tree-based algorithms (synthpop package in R) in the context of athlete monitoring data in sport, with the aim of providing an educational primer and discussion for potential application of these methods when exploring issues in the field sports and exercise sciences.

**Methods:**

The software package in R, synthpop, was used in seven simulation conditions applied to a professional football dataset, with varying model constraints. Classification and regression trees were used as the base model framework for each simulation. Metrics associated with both global utility (overall dataset similarity) and specific utility (specific research outcome similarity) were assessed on each simulation condition.

**Results:**

All simulation conditions demonstrated high levels of global utility. Additionally, simpler simulation conditions, which more closely resembled the analysis of the original dataset (simulation condition 1 and 2), provided higher specific utility than more advanced simulation conditions.

**Conclusion:**

To summarize, three types of models can be conceptualised for generating synthetic data: (1) models used for analysis of the original data (answering specific research questions), (2) models used to generate synthetic data, and (3) models that represent the true generation process for the original data. Misalignments in the specifications of these models might introduce biases that can compromise the utility of synthetic data no matter the purpose. As synthetic data do not constitute a direct replacement for real data from conceptual and empirical standpoints, we believe that researchers embracing this practice must include sufficient documentation concerning the synthetic data generation process purpose, the predictors and model used, and the potential boundary conditions for using the synthetic data in future investigations in sports and other fields.

**Supplementary Information:**

The online version contains supplementary material available at 10.1007/s40279-025-02221-6.

## Key Points

**Table:** 

A synthetic dataset’s goal should be clearly communicated. If the goal is to generate synthetic data generation that can be tested and analysed in specific ways, then an appropriate generation process should be selected that maps to the specific utility of the dataset.
Synthetic data should be accompanied by documentation of its generation process, including the predictors used, the model framework used for generation, and the potential limitations associated with how a generated dataset could be explored as a part of future research.
As a community, we should develop appropriate processes for improving transparency around synthetic data generation for open release. Additionally, sport researchers wanting to generate fit-for-purpose synthetic datasets should partner with relevant expertise (from data science and statistics).

## Introduction

Recently, there have been calls for increasing open science practices and rigor in sports science [1], including increased transparency and improved quality of evidence for greater impact and translation. One core goal of open science efforts is to facilitate *findable, accessible, interoperable, and reusable* (FAIR) data, study registrations, study protocols, analysis plans, and code [2]. For example, open science practices in sports sciences could circumvent common sample size issues by collating data from individual sports teams, single small organizations, or leagues. Likewise, these collated datasets could support research efforts that incentivize experimental and methodological rigor [1].

Barriers in sports science, particularly in high-performance environments, can prevent researchers from embracing an open and FAIR data framework owing to reasons that are beyond the researchers’ control. Data can sometimes be viewed as a commodity in sport given the potential for competitive advantage over other teams or clubs and possible commercial opportunities [3]. Moreover, potential data re-identification remains a substantial risk, stifling open science culture in sport research. Accordingly, in alignment with improving open science in sport, there is a need for the development of strategies allowing for basic demographics, exposure, and a minimal set of relevant measures to be shared in high-performance sports research, without sports clubs losing any competitive advantage and while protecting the identity of individual athletes [1].

Issues challenging open science and FAIR data principles are not unique to sport. In healthcare, electronic health records (or as a part of clinical experiments) contain highly sensitive information, and gaining access to these datasets can be costly and time-consuming [4]. Anonymization is one method to facilitate data sharing practices [5]. Another method is to create synthetic data [6]. Synthetic data resemble the data from the actual study but include (1) some differences so that individuals cannot be identified, and (2) enough similarities that the results closely resemble the results from the true data (i.e., have the same statistical outcomes as the original data). In this sense, individual observations will not be identical to the original data.

### What Is a Synthetic Dataset?

Synthetic data are data that have been generated, or in simpler terms simulated, using purpose-built mathematical models or algorithms, with the aim of solving data science tasks [7]. As an alternative form of statistical simulation, a synthetic data generation process leverages existing population data with the objective of exploring real-world situations by drawing samples from close-to-reality populations [8]. Synthetic data can facilitate open science practices (by making data open for secondary analyses) [9] and can be used for developing code or generating and testing hypotheses before deployment on real datasets or for the facilitation of training in handling complex medical data [10].

Synthetic data generation can use *process-driven methods* or *data-driven methods* depending on the objective [6]. *Process-driven* methods derive synthetic data from computational or mathematical models of an underlying physical data-generating process per se. These are typically based on physical laws or other mechanistic models describing the data generation process. The methods are generally useful if the underlying “true” or at least putative mechanisms underpinning synthetic data generation (i.e., rules or distributions) are known or can be accurately estimated. Examples include numerical simulations, agent-based modeling, and discrete-event simulations. Agent-based models have contributed to synthetic data generation in urban disaster research [11], and physics-based systems and numerical simulations have been used to generate synthetic data using information regarding fluid flow and solute transport in water resource research [12].

*Data-driven* methods are useful for generating synthetic data that accurately resemble some aspect(s) of a specific sample of data. The process uses generative models based on relationships observed within the original data without necessarily relying on a deep understanding of the mechanisms that generated it. Since our objective is to create shareable datasets, we always refer to *data-driven* methods when we use the term “synthetic data” in the rest of this manuscript. Data-driven synthetic data have received particular attention for over 30 years [13], with its utility being of strong interest in healthcare and medicine [6]. In the context of making previously observed data available, we attempt to preserve as much of the statistical patterns from the original data as possible. Real observations are replaced with synthetic observations, ensuring enough variation from the original data that individual data records do not reflect any one individual. These data-driven synthetic datasets can be implemented using a range of software packages (e.g., for R and Python) [8–10]. One R package, synthpop [14], is growing in popularity with applications in prenatal healthcare [15], cancer [9], and biobehavioral data contexts [16]. Recently, synthpop has made its way into sport science, with a demonstration on two open datasets [17] and the construction of a dashboard for synthetic data generation (https://assetlab.shinyapps.io/SyntheticData/).

In addition to this growth in the application of synthetic data, there is also interest in exploiting these synthetic datasets for new (or secondary) research explorations. This would involve leveraging the information embedded within synthetic datasets to gain new insights and generate new hypotheses that could be tested as a part of future studies. For example, Vaden et al. [18] synthesized neuroimaging, demographic, and behavioral data to (among other things) advance scientific discovery in neuroscience [18]. This desire to explore synthetic data is also common in other forms of clinical research, administrative data, and other longitudinal population-based studies [19].

### Present Study

Given the potential to improve open science and FAIR data principles in sport through the integration of synthetic data, and the construction of the relatively user-friendly synthpop package in R, and the application of synthpop in sport [17], our objective is to explore and scrutinize the process of generating representative synthetic data using a previously published athlete monitoring dataset [20]. The original dataset has been used in studies investigating the relationship between training load and injuries—an area that is featured in numerous publications [21, 22] yet not without methodological shortcomings and inconsistencies [23–26]. Creating open and shared synthetic datasets would enable the replication and reanalysis of previously collected data, allowing further exploration and investigation of previous studies’ methods.

Despite synthpop’s potential, generating a synthetic training load and injury dataset has challenges beyond the synthpop package and standard default settings, requiring careful thought regarding the specifications for how the synthetic data are generated. It is important to document these decisions and acknowledge the limitations and constraints of the resulting synthetic datasets [27]. Therefore, we discuss how to approach these decision points and how the decisions might affect the validity of analyses that are conducted using the synthetic data.

We began with the intention of sharing the synthetic dataset, but this is not a straightforward process. It is easy to generate synthetic data, especially when the accessibility for the generation of such datasets has been improved through the development of applications (such as the Shiny application made for sport researchers). We wanted to provide a picture of the challenges that scientists may face when making synthetic datasets, the expertise required, and the limitations associated with making synthetic data. This is to limit misuse and the proliferation of available datasets that are used beyond their limitations.

## Methods

### Synthetic Data Generation

#### Original Dataset

Fanchini et al. [20] examined prospective data collected from a sample of 34 professional football players on the same team over three consecutive competitive seasons. A comprehensive review of these data and the original research context is available in Supplementary Material S1. Data reuse and publication of the results were approved by the authors of Fanchini et al. [20] and aligned with the original ethical approval (ID #IRBMS062017001, as granted by the S.S.MAPEI Institutional Review Board, Italy). The original study adhered to the ethical standards outlined in the 1964 Declaration of Helsinki and its subsequent amendments. We revisited a synthetic dataset based on these data that was created for a subsequent study [28].

#### Variables to be Synthesized

Within the current study, each synthetic dataset to be generated contains five variables from the original data in Fanchini et al. [20]:

1. *WeekID*: A numeric variable specifying the week to which the observation belongs. WeekID ranges between 1 and 120 for each player. Each week must be a positive integer, i.e., the specific week in the testing period.
2. *PlayerID*: A dummy nominal variable outlining a specified player ID, ranging from 1 to 34.
3. *Acute Load*: A variable serving as a proxy for each player’s weekly training load (during the current week, or *T*).
4. *Chronic Load*: a variable serving as a proxy for each player’s 4-week chronic load for each week (from week *T*, through to the fourth week). Chronic load was uncoupled [29].
5. *Injury*: A binary variable indicating whether a player was injured during that week (yes = 1, no = 0).

#### Original Analysis

Fanchini et al. [20] explored the association between load and noncontact injury using generalized estimating equations (GEEs). The original model included a logit link function and exchangeable working correlation matrix, being selected on the basis of lower quasi-likelihood under the independence model criterion [30]. These specifications and outcomes were replicated for each synthetic dataset that was generated.

#### Synthetic Data Generation Process

We used the synthpop package in R to generate synthetic data [14]. A full description of synthpop and the processes for generating synthetic data using this package can be found in Supplementary Material S2. A range of different model frameworks for generating synthetic data (parametric and nonparametric) are available within synthpop. Given the lack of research conducted into generating and using synthetic data in sport, the default method of nonparametric generation, viz. classification and regression trees (CART), was used to create each synthetic dataset. Synthetic data generation from Fanchini et al. [20] involved using different combinations (and orderings) of the five variables [20] as predictors for synthetic data generation. These combinations were specified through a series of simulation conditions (see Sect. 2.1.5). For each simulation condition, 500 synthetic datasets were generated. We assessed whether each synthetic dataset resembled the characteristics of the original dataset. Metrics for assessing how well each dataset resembled the original dataset (that is, its *performance*) are discussed in more detail further (Sects. 2.2.1 and 2.2.2).

#### Simulation Conditions

We followed an exploratory approach to examine how synthetic data quality and properties can change across different simulation conditions (i.e., specifications for how and which variables are generated). Table 1 provides details of each simulation condition, including the variables being synthetically generated (*Y*_obs_) and the predictors used to generate those variables (*X*_obs_).

**Table 1:** Description of each simulation condition

	Variables for *X*_obs_	Variables for *Y*_obs_	Conditioning variables for synthetic generation^†^	Visit sequence of predictors	Description and rationale
Synthetic data for AL and CL					
Simulation condition 1 (base)	Injury; PlayerID	Acute load	{Injury*, PlayerID*}	Injury; PlayerID; Acute load; Chronic load	Predictors consisted of the same variables used in the original GEE analysis (i.e., PlayerID as the ID variable, injury as an independent variable, and the acute–chronic training load ratio (ACLR) being calculated using both AL and CL) Rationale: A simple set of specifications for synthetic data generation, designed to provide a dataset that reports similar outcomes to the GEE model applied to the original data
		Chronic load	{Injury*, PlayerID*, Acute load}		
Simulation condition 2 (Base_week)	Injury; PlayerID; WeekID	Acute load	{Injury*, PlayerID*, WeekID*}	Injury; PlayerID; WeekID; Acute load; Chronic load	The same predictors from condition 1 were used, with the addition of WeekID, entered after PlayerID Rationale: WeekID was added to the specifications of condition 1, to capture any temporal structures in the data across weeks. This was not possible with condition 1 specifications
		Chronic load	{Injury*, PlayerID*, WeekID*, Acute load}		
Simulation condition 3 (Time_Lag_1wk)	Injury; PlayerID; AL_Lag(1-step); CL_Lag(1-step)	Acute load	{Injury*, PlayerID*, AL_Lag(1-step)*, CL_Lag(1-step)*}	Injury; PlayerID; AL_Lag(1-step); CL_Lag(1-step); Acute load; Chronic load	WeekID was replaced in preference over two lagged variables, with each being one time step backwards for acute and chronic load, respectively Rationale: This allowed for any autoregressive trends in acute and chronic load (captured one time backwards) to be captured as a part of the synthetic data generation process
		Chronic load	{Injury*, PlayerID*, AL_Lag(1-step)*, CL_Lag(1-step)*, Acute load}		
Simulation condition 4 (Time_Lag_3wks)	Injury; PlayerID; AL_Lag (1-step); AL_Lag(2-step); AL_Lag(3-step); CL_Lag(1-step); CL_Lag(2-step); CL_Lag(3-step)	Acute load	{Injury*, PlayerID*, AL_Lag(1-step)*, AL_Lag(2-step)*, AL_Lag(3-step)*, CL_Lag(1-step)*, CL_Lag(2-step)*, CL_Lag(3-step)*}	Injury; PlayerID; AL_Lag(1-step); AL_Lag(2-step); AL_Lag(3-step); CL_Lag(1-step); CL_Lag(2-step); CL_Lag(3-step); Acute load; Chronic load	Used the specification template as condition 3, with the exception that three lagged variables (one, two, and three time steps backwards) for acute and chronic load were constructed and used as predictors for acute and chronic load Rationale: This was similar to condition 3, but testing whether temporal autoregressive structures exist further back in time than just the week before the current data point
		Chronic load	{Injury*, PlayerID*, AL_Lag(1-step)*, AL_Lag(2-step)*, AL_Lag(3-step)*, CL_Lag(1-step)*, CL_Lag(2-step)*, CL_Lag(3-step)*, Acute load}		
Synthetic data for AL, CL, and injury					
Simulation condition 5 (Time_Lag_Injury)	PlayerID; AL_Lag(1-step); AL_Lag(2-step); AL_Lag(3-step); CL_Lag(1-step); CL_Lag(2-step); CL_Lag(3-step)	Acute load	{PlayerID*, AL_Lag(1-step)*, AL_Lag(2-step)*, AL_Lag(3-step)*, CL_Lag(1-step)*, CL_Lag(2-step)*, CL_Lag(3-step)*}	PlayerID; AL_Lag(1-step); AL_Lag(2-step); AL_Lag(3-step); CL_Lag(1-step); CL_Lag(2-step); CL_Lag(3-step); Acute load; Chronic load; Injury	In condition 5, the same predictors were used as condition 4, with injury being added as a variable to be synthetically generated. Injury was placed at the end of the visit sequence, so that acute and chronic load could be used in the prediction of injury (in addition to other predictors) Rationale: Injury was added at the end of the visit sequence as a variable to be generated to allow for the acute and chronic load variables to be used in its generation. This was necessary given the direction of assumed relationship between acute and chronic load data (i.e., predictors) and injury in the original study (i.e., the use of the GEE model for assessing the effect of acute–chronic training load ratio on injury outcomes)
		Chronic load	{PlayerID*, AL_Lag(1-step)*, AL_Lag(2-step)*, AL_Lag(3-step)*, CL_Lag(1-step)*, CL_Lag(2-step)*, CL_Lag(3-step)*, Acute load}		
		Injury	{PlayerID*, AL_Lag(1-step)*, AL_Lag(2-step)*, AL_Lag(3-step)*, CL_Lag(1-step)*, CL_Lag(2-step)*, CL_Lag(3-step)*, Acute load, Chronic load}		
Simulation condition 6 (Injury_Time_Lag)	PlayerID; AL_Lag(1-step); AL_Lag(2-step); AL_Lag(3-step); CL_Lag(1-step); CL_Lag(2-step); CL_Lag(3-step)	Acute load	{PlayerID*, AL_Lag(1-step)*, AL_Lag(2-step)*, AL_Lag(3-step)*, CL_Lag(1-step)*, CL_Lag(2-step)*, CL_Lag(3-step)*}	Injury (random sample); PlayerID; AL_Lag(1-step); AL_Lag(2-step); AL_Lag(3-step); CL_Lag(1-step); CL_Lag(2-step); CL_Lag(3-step); Acute load; Chronic load	Injury was repositioned at the start of the visit sequence as a random sample, with all other variables being fit around injury. The remainder of the visit sequence stayed consistent with the above conditions Rationale: Injury was repositioned and entered as a random sample, owing to the computational cost of generating synthetic data noted in condition 5 (see Sect. 3). This was to test whether adding injury as a random sample could improve the time taken computationally to construct synthetic data
		Chronic load	{PlayerID*, AL_Lag(1-step)*, AL_Lag(2-step)*, AL_Lag(3-step)*, CL_Lag(1-step)*, CL_Lag(2-step)*, CL_Lag(3-step)*, Acute load}		
		Injury	Randomly sampled		
Simulation condition 7 (No_PlayerID)	AL_Lag(1-step); AL_Lag(2-step); AL_Lag(3-step); CL_Lag(1-step); CL_Lag(2-step); CL_Lag(3-step)	Acute load	{AL_Lag(1-step)*, AL_Lag(2-step)*, AL_Lag(3-step)*, CL_Lag(1-step)*, CL_Lag(2-step)*, CL_Lag(3-step)*}	AL_Lag(1-step); AL_Lag(2-step); AL_Lag(3-step); CL_Lag(1-step); CL_Lag(2-step); CL_Lag(3-step); Acute load; Chronic load; Injury	PlayerID was removed from the visit sequence, and injury was used as the final variable to be synthetically generated Rationale: The computational time to generate synthetic data improved substantially in condition 6, indicating that adding injury to the generation process as a variable to be generated did substantially increase the complexity of the generation process. This led to a compromise in condition 7, dropping PlayerID as a predictor to allow for injury to be generated, and predicted relative to all acute and chronic load-related variables
		Chronic load	{AL_Lag(1-step)*, AL_Lag(2-step)*, AL_Lag(3-step)*, CL_Lag(1-step)*, CL_Lag(2-step)*, CL_Lag(3-step)*, Acute load}		
		Injury	{AL_Lag(1-step)*, AL_Lag(2-step)*, AL_Lag(3-step)*, CL_Lag(1-step)*, CL_Lag(2-step)*, CL_Lag(3-step)*, Acute load, Chronic load}		

The first four conditions focus specifically on generating synthetic data for acute load and chronic load only. Conditions 5–7 involved the addition of injury as a variable to be synthetically generated in new datasets. In this table , *X*_obs_ are variables that are not synthesized while *Y*_obs_ are variables that are to be synthesized

*PlayerID* ID variable identifying each player, *WeekID* ID variable identifying the week of each observation, *AL_Lag(1-step)* variable based on one time step backward for acute load, *AL_Lag(2-step)* variable based on two time steps backward for acute load, *AL_Lag(3-step)* variable based on three time steps backward for acute load, *CL_Lag(1-step)* variable based on one time step backward for chronic load, *CL_Lag(2-step)* variable based on two time steps backward for chronic load, *CL_Lag(3-step)* variable based on three time steps backward for chronic load, *GEE* generalized estimating equation, *ACLR* acute–chronic load ratio

*Variable taken from original data and not synthesized

^†^Conditioning variables are displayed for the second scenario of calculating chronic load, where chronic load is treated as a variable independent of acute load. In scenario one, chronic load is calculated directly from the synthetic acute load

Simulation conditions 1–4 were simple in their design, generating synthetic data for only two variables, viz. acute load and chronic load, while “*fixing*” all other variables (but still allowing these variables to be used as predictors for acute load and chronic load). These simulation conditions aimed to preserve possible temporal structures of the original training load data, particularly information related to data patterns at the individual player (*PlayerID*) and repeated measures (WeekID) levels. These four conditions provided insight into how different predictor specifications change the properties of synthetic data generated from longitudinal athlete monitoring datasets—a common context in sport research.

In simulation condition 1 (Base), only PlayerID, acute load, chronic load, and injury were used as predictors, with these being the original variables involved in the GEE model in Impellizzeri et al. [28]. In simulation condition 2 (Base_week), WeekID was added as a predictor as one method for capturing temporal structures across weeks within each player. In condition 3 (Time_Lag_1wk), we created an autoregressive (i.e., time-lagged) variable based on one time-step backward for acute load (AL_Lag(1-step)) and chronic load (CL_Lag(1-step)), using the original acute load and chronic load data for lagged predictors. Similarly, in condition 4 (Time_Lag_3wks), we created three lagged variables, akin to an AR(3) model (1, 2, and 3 weeks backward for acute load (AL_Lag(1-step); AL_Lag(2-step); AL_Lag(3-step) and chronic load (CL_Lag(1-step); CL_Lag(2-step); CL_Lag(3-step)). These newly created variables were included as additional predictors for synthetic data generation for different simulations (Table 1).

Simulation conditions 5 and 6 (Time_Lag_Injury; Injury_Time_Lag) used some of the specifications to handle the temporal structures of acute load and chronic load across simulation conditions 1–4 while generating new synthetic injury locations in the dataset. This was necessary since the date and location of an injury could potentially re-identify an athlete, presenting possible privacy concerns. Simulation condition 7 (No_PlayerID) was identical to time_lag_injury but with the removal of the variable PlayerID.

#### Scenarios for Creating Synthetic Chronic Load

Across all seven simulation conditions, two scenarios were tested for generating synthetic chronic load data. In the first instance, the chronic load was *independently generated* (scenario CL_independent) and treated as an independent variable to be synthesized. In the second instance, the synthetic chronic load was *calculated from the synthetic AL* (scenario CL_calculated) (calculated identically to the original data across a 4-week period). This comparison was performed because of the mathematical coupling that exists between acute and chronic training load [29], which is a salient yet problematic characteristic compromising this area of research.

### Metrics for Assessing Synthetic Data

There are two broad ways to assess the quality, or utility, of synthetic data. Firstly, the *global utility* assesses the overall similarity of the synthetic data to the original data across all specified variables, independent of any specific research question [31]. Essentially, this assesses whether the different variables’ value distributions match across the original and synthetic datasets. However, even if the overall marginal distributions are similar, any relationship *between* variables in the synthetic data may differ from those in the original set if the model choices for synthetic data generation did not match the true data generating process of the original observed data. If the relationships differ, analyses related to specific research questions would likely be biased. Therefore, another type of metric, viz. the *specific utility*, assesses the ability of the synthetic data to replicate the original dataset’s answer to a specific research question or outcome [31].

#### Global Utility

Three common global utility metrics use a prediction model (the default in synthpop being a logistic regression, which was used in the present study) to discriminate between the source and synthetic datasets. These metrics employ a propensity score [31, 32], which represents the probability of each observation being either real or synthetic (0 or 1, respectively). *High* global utility implies that the datasets are indistinguishable. From this logistic regression model, the propensity score-weighted mean squared error (pMSE); the standardized mean squared error (s-pMSE), which is *z*-scored relative to a null distribution; and the percentage of the observations correctly predicted over 50% (PO50) were derived and used as global utility metrics [31, 33]. Lower values for each metric imply better global utility (Supplementary Material S3).

#### Specific Utility and Additional Metrics

Specific utility refers to measures evaluating how well results from synthetic data-based analyses replicate results from original data-based analyses. In the present study, we investigated specific utility by replicating the GEE outcomes from the work of Impellizzeri et al. [28]. For this, we calculated the mean absolute errors (MAE) of (1) the GEE parameter estimates, (2) the GEE standard errors, and (3) the GEE *p*-values for acute–chronic workload ratio at 4 weeks by comparing 500 synthetic datasets’ results with the original dataset’s (with a log odds model being used). Lower values for each of these metrics imply greater specific utility (i.e., greater closeness of the synthetic data to the original data), and these metrics, along with some of secondary interest, are explained in detail in Supplementary Material S3. In addition to measures of specific utility, computation time was measured for each synthetic dataset being constructed. An MAE between the original and synthetic values of acute and chronic loads was calculated to understand better whether temporal training load trends were retained at the individual player level.

Finally, for simulation conditions 1–4, the variability of the underlying data generation process within each simulation condition was compared across all synthetic datasets generated. This involved comparing synthetic datasets as a series of adjacent pairs (i.e., dataset 1 and dataset 2, dataset 2 and dataset 3, and so on). For each set of adjacent pairs, the same metrics were calculated for each pair of synthetic datasets (i.e., the MAE was calculated using the absolute error between pairs of synthetic datasets), providing an indication of whether a simulation condition was likely to be more inconsistent, regardless of its performance for specific utility when assessed relative to the original data. The results for this test are provided in Supplementary Material S4 and briefly discussed therein relative to the general results of specific utility for these four simulation conditions. All materials and code for running through these demonstrations is available at https://github.com/johnwarmenhoven/SynthData_in_Sport/tree/main.

## Results

Descriptive statistics for the *global utility* and *specific utility* metrics are presented in Table 2.

**Table 2:** Results for measures of global utility, computation, and specific utility for the first four simulation conditions, focused on generating synthetic data for the two training load variables (acute and chronic) in simulation conditions 1–4 and the two training load variables and injuries in simulation conditions 5–7

		Synthetic training load data simulations						
		Base (1)	Base_Week (2)	Time_Lag_1wk (3)	Time_Lag_3wks (4)	Time_Lag_Injury (5)	Injury_Time_Lag (6)	No_PlayerID (7)
		Mean (SD)	Mean (SD)	Mean (SD)	Mean (SD)			
Global utility								
	pMSE	< 0.01 (< 0.01)	< 0.01 (< 0.01)	< 0.01 (< 0.01)	< 0.01 (< 0.1)	–	< 0.01 (< 0.01)	< 0.01 (< 0.01)
	s-pMSE	1.2 (0.44)	1 (0.37)	1.14 (0.43)	0.85 (0.27)	–	0.86 (0.29)	0.93 (0.32)
	PO50	0.74 (0.46)	0.65 (0.47)	0.57 (0.45)	0.45 (0.39)	–	0.49 (0.41)	0.48 (0.43)
Specific utility (MAE)								
Chronic load simulated as independent variable	GEE estimate	0.37 (0.27)	0.37 (0.26)	0.48 (0.29)	0.75 (0.32)	–	0.91 (0.42)	0.61 (0.41)
	GEE SE	0.1 (0.06)	0.11 (0.07)	0.11 (0.07)	0.14 (0.07)	–	0.09 (0.07)	0.14 (0.09)
	*p* Value	0.03 (0.07)	0.12 (0.20)	0.36 (0.28)	0.57 (0.27)	–	0.49 (0.29)	0.39 (0.31)
Chronic load calculated from synthetic acute load	GEE estimate	0.33 (0.27)	0.75 (0.40)	0.83 (0.35)	1.16 (0.34)	–	0.93 (0.44)	1.05 (0.50)
	GEE SE	0.13 (0.08)	0.13 (0.07)	0.12 (0.07)	0.1 (0.06)	–	0.14 (0.08)	0.15 (0.08)
	*p* Value	0.11 (0.17)	0.48 (0.28)	0.55 (0.27)	0.49 (0.29)	–	0.50 (0.29)	0.47 (0.30)
Acute load	Observation level	462.57 (6.82)	365.65 (6.40)	330.59 (6.10)	295.02 (5.62)	–	–	–
Chronic load, simulated	Observation level	272.26 (4.42)	208.28 (3.82)	142.12 (2.63)	126.03 (2.50)	–	–	–
Chronic load, calculated	Observation level	257.05 (5.77)	196.81 (4.49)	194.92 (4.35)	180.86 (4.19)	–	–	–
Computation	Time (s)	0.08 (0.02)	0.1 (0.03)	0.15 (0.04)	0.21 (0.02)	22.16 min	0.1 (0.03)	0.15 (0.04)

*MAE* mean absolute error, *pMSE* propensity score mean squared error, *s-pMSE* standardized propensity score mean squared error, *PO50* percentage of the observations correctly predicted over 50%, *GEE* generalized estimating equation, *SE* standard error, *s* seconds

### Global Utility

The global utility was high across all simulation conditions, with the largest values of pMSE and s-pMSE across all simulations and simulation conditions being less than 0.01 and 1.20, respectively, indicating a strong level of overall similarity between the original and synthetic datasets. Measures of s-pMSE improved as more temporal predictors were added across simulation conditions 1–4, with these remaining consistent with conditions 5–7, where temporal predictors were also used as a part of the generation process.

### Specific Utility

As mentioned in Impellizzeri et al. [28], the analyses with original data showed effects compatible with higher injury risk for ACWR (4 weeks) (log-odds: 0.90, SE: 0.33, 95% CI 0.24–1.55, *p* < 0.01).

Across simulation conditions 1–4, where only acute load and chronic load variables were synthetically generated, the Base model (1) provided the best overall specific utility relative to the MAE of the GEE parameter estimate (GEE estimate MAE = 0.37 for simulating synthetic chronic load and 0.33 for calculating synthetic chronic load). It also provided the lowest MAE of the GEE *p*-values (MAE = 0.03 for simulating synthetic chronic load, MAE = 0.11 for calculating synthetic chronic load), indicating that simulating synthetic chronic load provided accurate replication of outcomes in the original GEE analysis. Despite this, the MAE for the GEE SE was poorer than for other simulation conditions when the chronic load was calculated from the synthetic acute load.

These trends for specific utility outcomes to be poorer when temporal predictors were used in the synthetic data generation process were also shown across simulation conditions 5–7, where temporal predictors were used. Given that simulation condition 1 most closely resembled specifications similar to the original GEE model, this suggests that, as the synthetic data generation model moves further away from the GEE model, specific utility outcomes were likely to be poorer.

### Additional Metrics

#### MAE of Acute Load and Chronic Load

These metrics were only calculated for simulation conditions 1–4. The results when we preserved the overall temporal structures were the opposite of the GEE results. The Base simulations had the worst (i.e., highest) MAE for both acute load (MAE = 462.57) and chronic load (MAE simulated chronic load = 272.26; MAE calculated chronic load = 257.05). Conversely, the simulation where we included time-lag variables for the previous 3 weeks (Time_Lag_3wks, simulation 4) provided the best outcomes for MAE of both acute load and chronic load (for both simulated and calculated chronic load scenarios).

This indicated that the inclusion of autoregressive terms improved the ability of the synthetic data to hold temporal characteristics of acute load and chronic load at the individual player level.

**Fig. 1:**
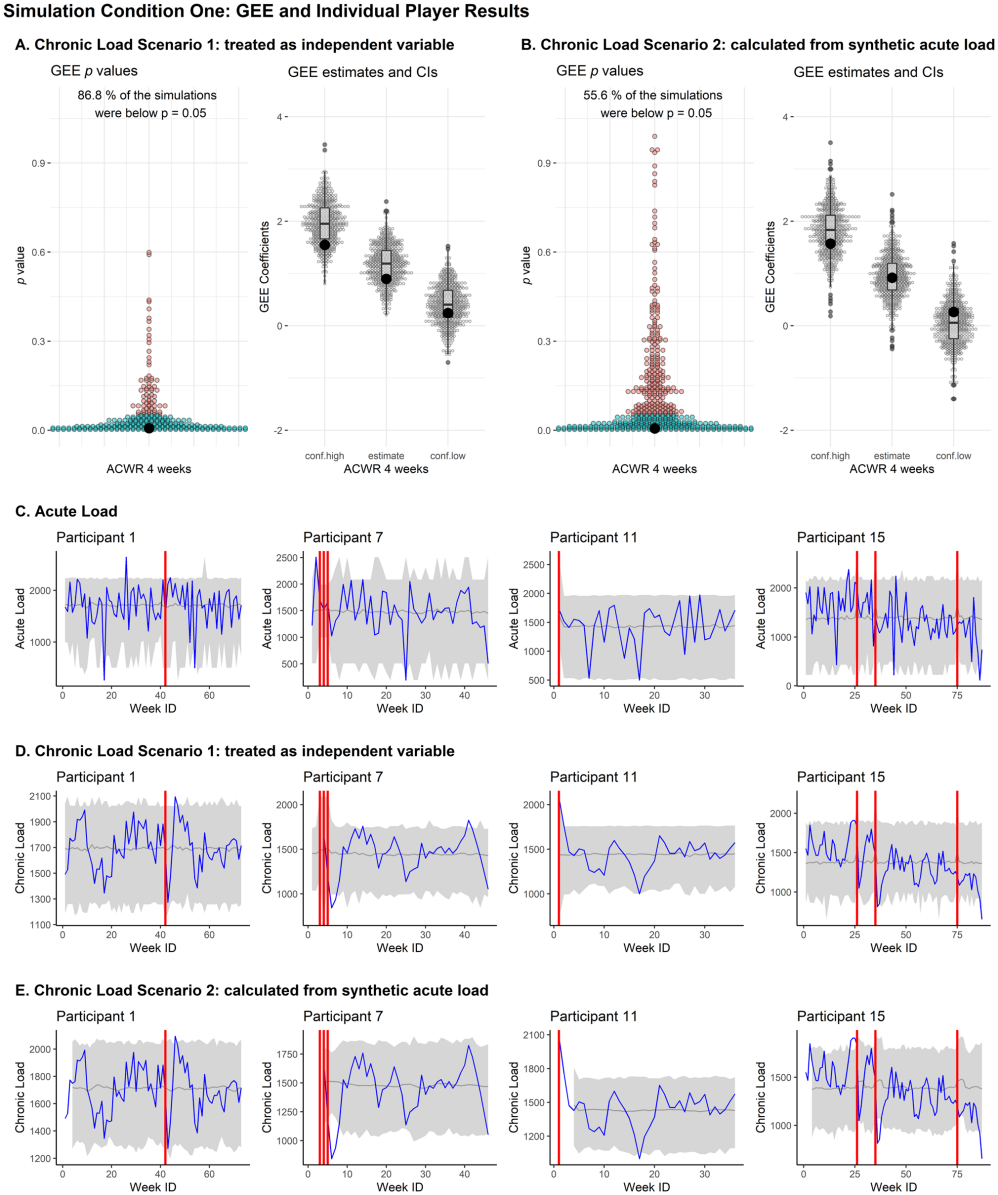
The above figure demonstrates GEE outcomes and individual player data for simulation condition 1. **A** GEE results from scenario one for calculating synthetic chronic load. These are GEE *p* values, GEE estimate, and GEE upper (conf.high) and lower (conf.low) coefficients for the 500 simulated synthetic datasets. The same outcomes from the original dataset are shown by the large black dot in each plot. **B** Same results as **A**, but for the second scenario for constructing the synthetic chronic load. **C** Examples of acute load for four exemplar participants. The original data trace is displayed in blue, the original injury locations are displayed as vertical red lines, and 95% percentiles across the 500 synthetic data simulations are displayed in grey. **D** Examples of the same four exemplar participants for scenario 1 of calculating chronic load. Properties of these graphs are the same as in **C**. **E** examples of the same four exemplar participants for scenario 2 of calculating chronic load. *WeekID* ID variable identifying the week of each observation; *GEE* generalized estimating equation, *ACWR* acute–chronic workload ratio, *conf.high* upper limit of confidence interval, *conf.low* lower limit of confidence interval

**Fig. 2:**
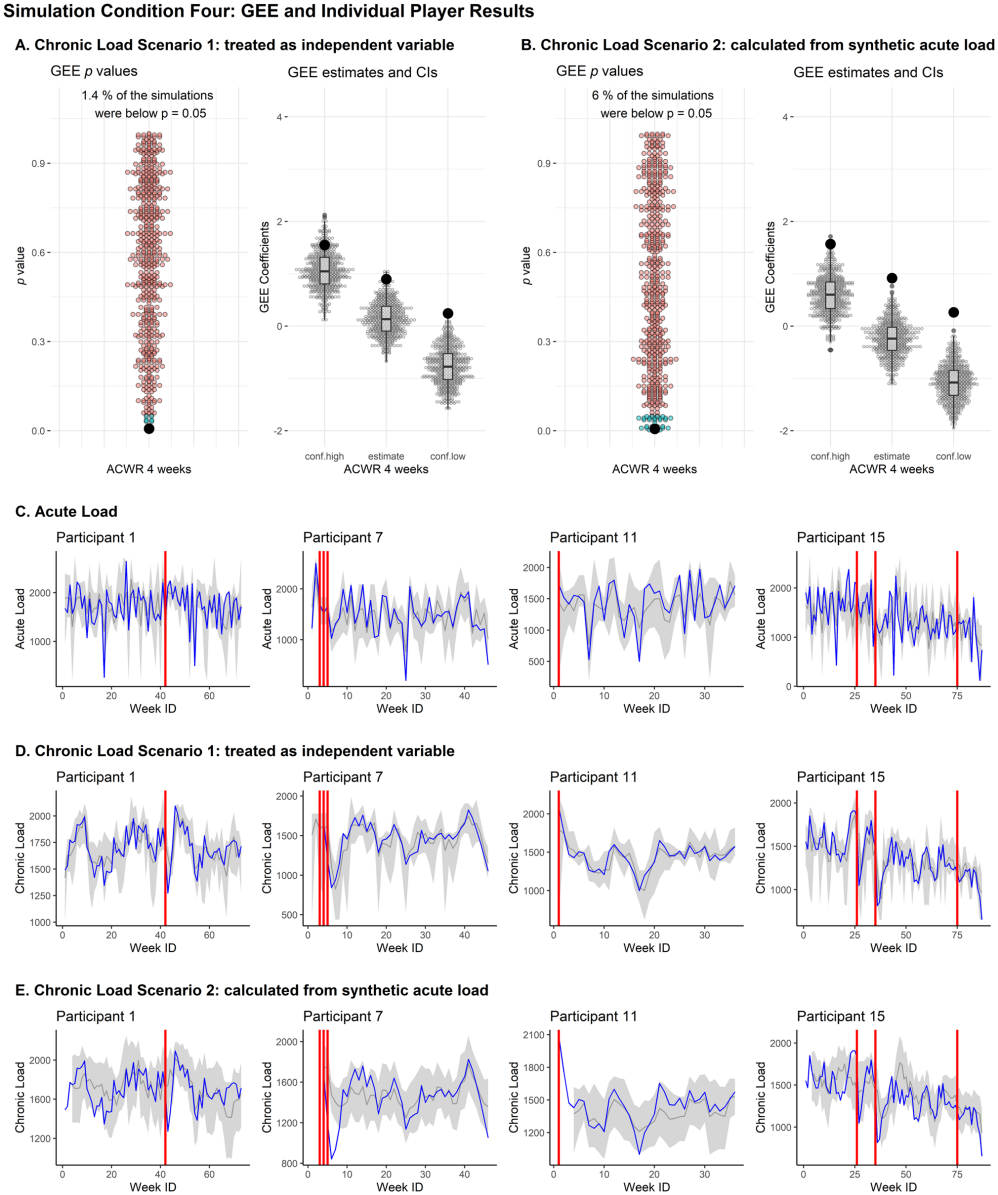
The GEE outcomes and individual player data for simulation condition 4. **A** GEE results from scenario 4 for calculating synthetic chronic load. These are GEE *p*-values, GEE estimate, and GEE upper (conf.high) and lower (conf.low) coefficients for the 500 simulated synthetic datasets. The same outcomes from the original dataset are shown by the large black dot in each plot. **B** The same results as in **A**, but for the second scenario for constructing synthetic chronic load. **C** Examples of acute load for four exemplar participants. The original data trace is displayed in blue, the original injury locations are displayed as vertical red lines, and 95% percentiles across the 500 synthetic data simulations are displayed in grey. **D** Examples of the same four exemplar participants for scenario 1 of calculating chronic load. Properties of these graphs are the same as in **C**. **E** examples of the same four exemplar participants for scenario 2 of calculating chronic load. *WeekID* ID variable identifying the week of each observation, *GEE* generalized estimating equation, *ACWR* acute–chronic workload ratio, *conf.high* upper limit of confidence interval, *conf.low* lower limit of confidence interval

This is demonstrated in Fig. 1, where large confidence bands (across the 500 synthetic datasets) can be seen around four exemplar players for simulation condition 1, despite the positive results in replicating GEE outcomes that were consistent with the original dataset. Figure 2 is representative of the same four players in simulation condition 4 where three autoregressive variables were used for synthetic data generation. The confidence bands across the 500 synthetic datasets have narrowed substantially, but with much poorer GEE replication outcomes.

**Fig. 3:**
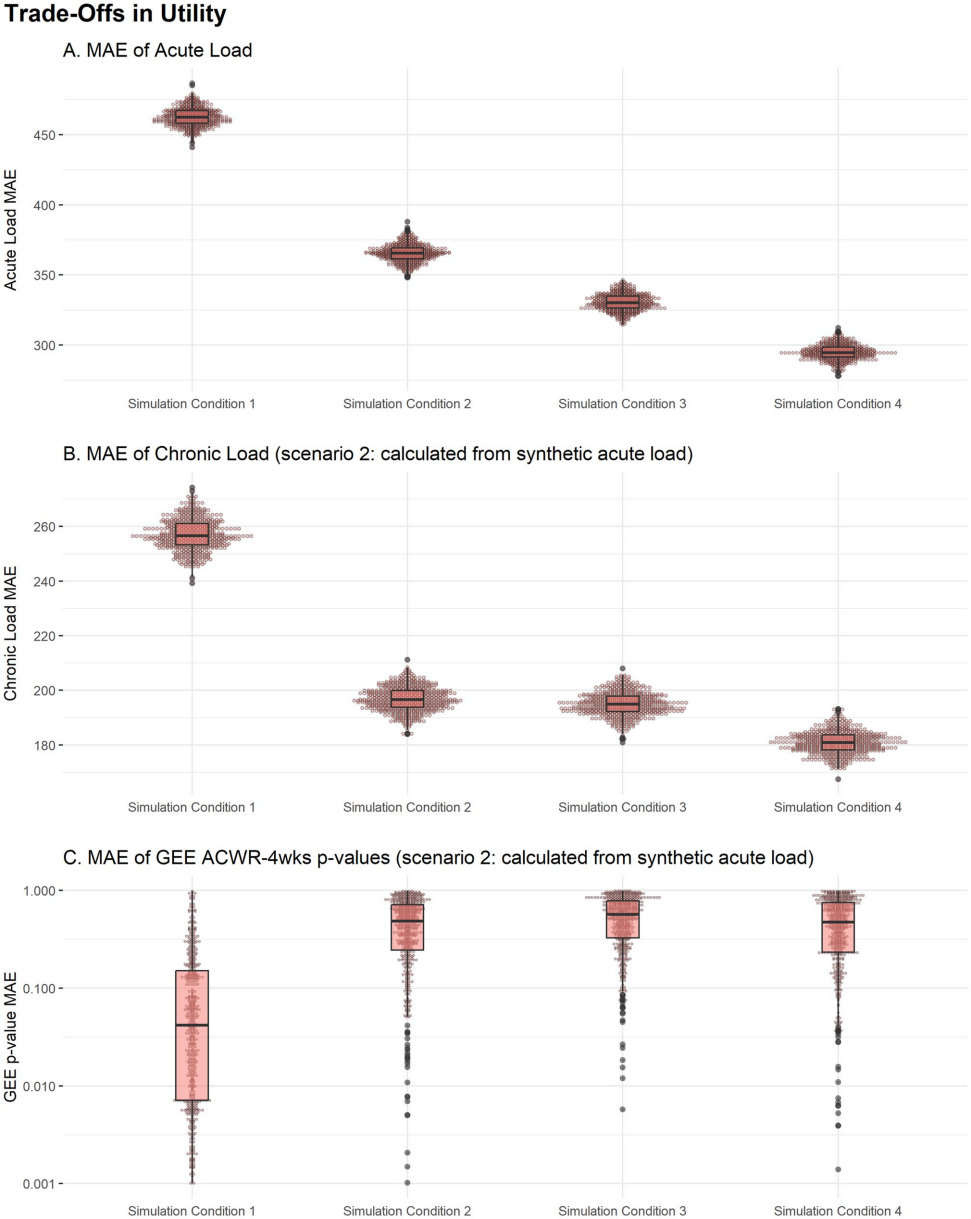
A demonstration of the trade-off between levels of error between the synthetic data and original data for replicating the original acute (i) and chronic (ii) variables for each player, and being able to replicate the same statistical outcomes (log-transformed *p* values) from the GEE model (iii). *MAE* mean absolute error, *GEE* generalized estimating equation, *ACWR* acute–chronic workload ratio

As more temporal predictors were added across simulation conditions 1–4 (Fig. 3), the GEE outcomes (i.e., specific utility) were poorer even though the MAE of the acute and chronic load variables improved.

#### Computation Time

The computation time was relatively consistent across all simulation conditions, ranging on average from 0.9 to 0.26 s per generated dataset, demonstrating high computational feasibility for generating many datasets and running testing across all simulation conditions. The only simulation condition not deemed feasible was when we added Injury as the final simulated variable in the time-lag simulations (simulation condition 5), which required 22.16 min to generate a single dataset; for this reason, we did not evaluate 500 replications and subsequently do not report on global and specific utility metrics across the 500 replications in Table 2. This is discussed further in the “Sect. 4.”

## Discussion

The current study serves as an educational primer exploring the strengths and limitations of using *data-driven* synthetic datasets to address open science and FAIR data principles in sports and exercise sciences. Through a series of simulation conditions, we highlight important considerations for a typical data context in sports and demonstrated how to assess and interpret the results. When the synthetic data generating process more closely aligned with the original GEE model in terms of the predictors used to generate synthetic data (i.e., simulation condition 1, Base), the synthetic data performed well at replicating GEE outcomes and thus provided better specific utility for the GEE-related research question. However, as the synthetic data generation process moved further away from the GEE model through the inclusion of temporal predictors and more closely mimicked the original data from individual players over time, the ability of the synthetic data to replicate GEE outcomes became poorer. Given this divergence in the specific utility across simulation conditions and the apparent ease of implementing packages such as synthpop, researchers need to understand a synthetic dataset’s characteristics and potential constraints to use it properly.

### Consideration 1: How Synthetic Data are Generated Predicates for What They Can be Used

As noted by Snoke et al. [31], results from models applied to synthetic data will only align with the same results applied to the original data if the models used to synthesize the data correspond to those that generated the original data. Our results confirm and illustrate that, if synthetic data are shared for reproducibility purposes alone, they will likely yield appropriate results if the model employed for generating the data is aligned with the research question and analytical models used in the original study. However, if the synthetic data are shared for additional exploration beyond the research questions and analytical models used in the original study, they will likely yield results that could be inconsistent with the original data. This is shown in the current investigation across the first four simulation conditions through replication of the same outcomes from the GEE model originally applied to the data in Fanchini et al. [20] serving as the test of specific utility for this study. Simulation conditions 1–4 were simple in their design, generating synthetic data for only two variables, viz. acute load and chronic load, while *fixing* all other variables (but still allowing these variables to be used as predictors for acute load and chronic load).

The synthetic data from the Base simulations aligned most closely with the original GEE analysis. This was because the data generation specifications aligned most closely to the original GEE model, using the same predictors for the synthetic data generation as the original GEE, despite leveraging a different generation framework. More specifically, we generated the synthetic data using classification and regression trees, and PlayerID was incorporated as a predictive variable in the generation process rather than an identification variable in the GEE. Although the majority of the specific utility outcomes from the GEE were best with the Base model, any temporal trends present within individual players’ load trajectories were lost in the Base simulations (large percentile bands in Fig. 1 for the four exemplar players).

As temporal variables were added across the next three simulation conditions (Base_week, Time_Lag_1wk, and Time_Lag_3wks), replication of GEE outcomes became poorer, but replication of the original temporal trends of acute load and chronic load improved (Fig. 3). This illustrates that, as more temporal predictors were added to the synthetic data generation process, the ability to replicate similar GEE outcomes became less possible. A simple explanation for this is that, if the GEE is assumed to be the process that generated the original data, the Base simulation condition most closely aligns to the specifications of the GEE and it will most likely provide outcomes consistent with those found in the original GEE. As more temporal predictors are added across the next three simulation conditions, the data generation process moves further away from the GEE model used in the original study.

The specifications of a synthetic data generation process (i.e., predictors used to generate data) govern what *can* be explored in a synthetic dataset. The Base simulation would allow for accurate replication of the original GEE analysis across the majority of the 500 datasets but would likely provide erroneous results if an independent research group used a different analytical approach to test the temporal characteristics of training load data leading up to the injury. Conversely, Time_Lag_3wks provides far fewer datasets that show comparable statistical outcomes to the original GEE analysis, but this condition provides 500 datasets that better represent the participant-level temporal trends embedded in the original data if temporal analyses were desirable.

The take-home message is that researchers must clearly state (1) how the data were synthetically constructed (i.e., which predictors were used to generate the synthetic data), (2) the limitations of any released synthetic datasets, especially in terms of how they *should* be explored given the constraints of the synthetic data generating model, and (3) the global and specific utility metrics used to evaluate the generated synthetic data, providing a rationale and justification for each metric. This will explicitly clarify what people can expect from using synthetic data once they are made open and what is possible with their use.

### Some Variables Should Not be Synthetically Generated

In the present study, two scenarios were used in each simulation condition to construct synthetic training load data. In the first scenario, synthetic chronic training load data were simulated from the data generation model, assumed to be independent of acute load. This relied on modeling conditional distributions to simulate new observations of synthetic chronic training load data. In the second scenario, chronic training load was derived directly from the new synthetic acute training load data, as an average of acute training load across a 4-week period. Except for the Base simulation, specific utility outcomes from the GEE models (i.e., the estimated MAE between synthetic and original GEE analyses) were better when chronic load was viewed as an independent variable. Additionally, simulating chronic load independently had a lower MAE compared with calculating chronic load from acute synthetic load, across the first four simulation conditions. Despite the apparently promising outcomes when chronic load is simulated, these synthetic datasets show undesirable statistical properties because they ignore the inherent mathematical deterministic relationship let alone the coupling between acute load and chronic load. When chronic load is simulated directly, this deterministic relationship no longer holds, and this may introduce important biases when conducting analyses or making inferences from the results. These findings also indicate that errors in constructing newly derived variables from the synthetic data may propagate forward. This should be explored further in more detail when derived variables are of interest for specific research questions. As a general guideline, if there are variables that are derived from other variables (i.e., chronic load in the current study), it is best practice to derive these new variables from the synthetic simulated variables (i.e., synthetic acute load in the current study). This allows for the construction of derived variables that are synthetic and also mathematically plausible relative to the variables from which they were derived.

The take-home message is that researchers should preserve mathematical (or deterministic) relationships that exist between variables when generating synthetic data, especially if independent researchers will be using the synthetic data for exploration. Researchers should also explore how errors are propagated across the newly derived variables of interest.

### Computational Burden Versus Accuracy

We included simulation conditions 5–7 given that the timing at which injuries occurred (i.e., specific WeekID location) could potentially be used to identify athletes. Other datasets may have similarly identifiable information, especially when individual teams are analyzed, and players have a public profile and injury data are public information. Thus, it was desirable to test circumstances where injury time was synthetically generated in addition to the two training load variables. This immediately led to computational problems for simulation Time_Lag_Injury—a single run of synthetic data took more than 22 min to complete. As a result, 500 simulations would take the same standard computer close to 1 week to finish computation before results could be inspected. For all intents and purposes, this is impracticable for most practitioners, scientists, and researchers aiming to test the generation of synthetic data using a set of conditions with computational demands similar to Time_Lag_Injury simulations across a range of datasets.

The length of time required for simulations could have occurred owing to the number and type of predictors used or the number of variables generated. The synthpop documentation indicates that there may be some difficulty when using predictors that have more than 20 factor levels, or when more than 12 variables are being used for synthesis [34]. In the current study, PlayerID had 34 factor levels and WeekID had up to 120 factor levels. PlayerID was used as a predictor in Time_Lag_Injury simulations. It is possible that the addition of another variable to synthesize may have led to generation models (through CART) that were difficult to build given the depth of factor levels in the predictors.

To address this issue, we added injury as a random variable to be generated in Injury_Time_Lag simulations (condition 6), with the synthetic training load data being refit around the locations of the random sample of injury locations. This was more computationally efficient but was less consistent with an intuitive understanding of the causal relationships between load and injury outcomes. Within the iterative synthpop process, data used as a random sample must be entered into the synthesis process first, indicating that injury times were specified before the synthetic training load data. However, the true data generation process is that both (1) training load causes injuries, and (2) injuries can lead to a reduction in training load later on (0 load in the case of a time-loss injury). If one is interested only in injury prediction using synthetic data generated this way, there is no bias relative to the direction of the effect between injury and load. However, if one is interested in causal effects, these temporal effects must be accounted for in the synthetic data or else biases may be introduced depending on the research question. In the context of the present study, this was included as a simulation condition solely to demonstrate the possibility of adding variables as a random sample starting point, and then fitting other variables around this starting point as a means of demonstrating better computational efficiency.

If it was still desirable to have injury entered after training load (insinuating that load leads to injury), it would be necessary to simplify the model for synthetic data generation, owing to the computational time issues with having injury positioned later in the variable visit sequence. To explore this issue, PlayerID was dropped as a predictor for synthetic data generation in the No_PlayerID simulations (condition 7). This reduced the number of variables with many factors, making it much faster to produce 500 datasets. These datasets’ specific utility was also better than Injury_Time_Lag simulations (Table 2). This comes at a cost: no player information or week information was used in the predictions, so the synthetic data will not reflect any likely trends for whether injuries will occur for specific players or weeks in the season. Despite this, No_PlayerID simulations preserved the relationships between the original training load and injury and the temporal autocorrelations between training load and injury (i.e., training load changes in the lead into an injury). Thus, the locations of these injuries relative to training load are “*fictitious*” within each player but provide a possible avenue for further exploration of temporal trends and injury outcomes.

The take-home message is that, when constructing synthetic datasets, there may be a compromise between computational feasibility and accuracy in capturing the relationships between variables in a dataset. Sport practitioners, scientists, and researchers need to think carefully about which relationships should be preserved in synthetic data and whether compromises are required owing to computational costs. Information regarding these relationships should be clarified and provided in documentation for future users of the synthetic datasets.

### Improving Transparency with Synthetic Data Generation

#### Documentation That Accompanies the Synthetic Data

The present study used the common method of sequential tree-based methods to construct data for each variable in a synthetic dataset [35, 36], where a variable is synthesized by using the values earlier in the sequence as predictors. As such, sequential synthesis processes are similar to modeling multiple outcome variables using classifier chains (i.e., assigning observations to more than one classification for a given variable) [37] and regressor chains (i.e., predicting a continuous value, or regression output, for a specific label independently) [38]. This is different from deep learning methods for generating synthetic data (e.g., generative adversarial networks [39]), which require very large datasets [6]. Sequential tree-based methods tend to work well for smaller datasets, such as traditional clinical trial datasets with heterogeneous variable types [40]. Providing information on the model framework used, a rationale for its selection and any testing of other model frameworks is desirable to ensure transparency around synthetic data generation. Additionally, future users of open synthetic data will need access to the data-generating model (and associated software and code) to evaluate the synthetic data and know which variables can be appropriately analyzed [41]. As the possible development and use of synthetic data grows in sports medicine and science, it will become increasingly important to develop standards regarding the construction, utility and reporting of synthetic datasets, for consistent use across journals. This is necessary for providing clarity around what should or should not be done with a given synthetic dataset. Such guidelines should leverage current provisional guidelines for creating synthetic data [42], existing domain knowledge on best practice for synthetic data generation [7], and domain-specific knowledge for the research field being explored.

#### Providing Multiple Synthetic Datasets

The synthpop documentation [14] encourages users to assess model performance across many synthetic datasets to determine whether the model for the synthetic data sufficiently captures salient features of the real data. In many instances using synthpop, synthetic data were deemed accurate and reflective of the relationships present in the original data [9, 15, 16]. These studies, however, evaluated only one or two synthetic datasets per context. Sharing such a small number of synthetic datasets is likely insufficient for truly understanding whether a synthetic generation process was well suited to the underpinning goals of a simulation study or a study releasing a particular dataset.

Releasing many synthetic datasets rather than just one synthetic dataset provides one avenue for testing exploratory research questions on synthetic data. If many datasets are released and estimates from new models applied to these datasets show consistent outcomes when the results are pooled, this may be indicative of a new trend captured in the original data [18]. However, there are two challenges. First, this would need to be verified by the original data as it could simply reflect bias arising from synthetic data model misspecification. Second, there is a trade-off in the benefits of generating many datasets. Some scientists and researchers may want to release more synthetic datasets to account for larger amounts of variance introduced into the synthetic data during different data generation processes [43]. This is relevant in the current study given the contrasting results of data generation processes having higher specific utility and more variability in their data generation processes (Supplementary Material S4). Generating more datasets to account for the heterogeneity of a data generation process is problematic though, given that observations across multiple synthetic datasets can be used to refine guesses on the original data [36, 44], making athletes potentially re-identifiable, defeating the point of using synthetic data in the first place. In these circumstances, careful consideration must be given to balancing the benefits of releasing multiple synthetic datasets, relative to the risk of re-identification for specific variables. Additionally, if the data generation process has a higher level of heterogeneity and only a small number of synthetic datasets are produced, it is possible that an outlier dataset may be released. Such a dataset could lead to inappropriate inferences being drawn on synthetic data, further reinforcing the care that should be taken with selecting and releasing synthetic datasets openly.

The take-home message is that any synthetic data released publicly must be accompanied by documentation outlining its possible use, the processes underpinning its generation and software or code for how it was generated (for an example of this, see Supplementary Material S5). If researchers want to avoid inappropriate inferences, conclusions, and recommendations, they should also test their model frameworks for synthetic data generation across multiple datasets and data contexts to better understand synthetic data generation processes. It is important for future development, research, and practice within the statistics and data science communities to focus on establishing guidelines and transparency for the use of synthetic data. This will assist scientists who are wanting to generate or utilize the potential of synthetic data.

#### Using Synthetic Data to Ensure Transparency of Code

One major benefit of software packages such as synthpop is the relative ease of generating synthetic replicates of a dataset (i.e., with a single line of code). This may be appealing for making a relatively fast dataset that has the same architecture and general characteristics as the original data. Synthetic data that are generated quickly and using default settings in synthpop may still be useful to supply in addition to manuscripts for checking the code that authors have developed for their analysis. In this sense, synthetic data can only be used for this purpose and not for any exploration or verification of original results. If researchers chose to use synthetic datasets in this way to permit verification of code, it would be sensible to include a report (i.e., in Markdown or otherwise) of the analytical outcomes from applying the code to the synthetic dataset, so that reviewers or readers know what to expect the outcomes of code applied to a synthetic dataset will look like.

### Limitations

Although this is one of the first studies to explore the potential of synthetic data in sport, results from the present study carry some limitations. Only one method of synthetic data generation was explored in the present study, i.e., a sequential tree-based approach for generating new synthetic variables. Synthpop has an array of other parametric and nonparametric model frameworks that were not trialed in this study. Beyond the model frameworks available in synthpop, there are several other methods that can be used to construct synthetic data, including mixture of product of multinomials (Mom), categorical latent Gaussian processes (CLGP), and generative adversarial networks (GAN) [6]. GANs could be of potential utility in future research for datasets similar to the present context, given their demonstrated application with time-series and “monitoring” datasets [45]. Similarly, agent-based models (ABMs) may also help evaluate unseen scenarios with the data context, provided we have a strong understanding of the causal relationships between variables [46]. Future research should explore the potential of other such model frameworks across different data contexts, but it should be stated that use of these alternative models without prior validation poses obvious risks.

There are some potential limitations to using the synthpop package’s sequential tree approach. The “*visit.sequence*” and variable ordering used when applying a sequential tree-based generation process can lead to greater computational issues when variables with a larger range of values are synthesized first [42, 47] (particularly those with continuous domains or many factors). Within a sequential generation process, the synthesized values will likely have low utility if the preceding variables are weak predictors of subsequent variables. Further, synthesis errors will propagate, and potentially be amplified, through the chain [48]. Some authors have tried to minimize error propagation by modeling variable dependence or using algorithmic approaches such as particle swarm optimization for identifying variable permutations that ensure consistent data utility [48]. The present study did not consider these more complex factors and how they may have affected the data generation process across all seven simulation conditions.

There are also potential limitations regarding the hierarchical (i.e., panel data) structures within the original dataset used for synthetic data generation. One example relates to using an alternative synthetic data generation method (i.e., differential privacy) for identifying input features for “machine learning” methods’ synthetic algorithms. This showed limited usability for more complex datasets with non-independent and identically distributed data (i.e., when hierarchies/repeated measures are present in the data) [49]. In the current context, the dataset’s complexity, resulting from the combination of repeated measures and independent variables, may have posed a challenge for standard CART processes to model and construct synthetic data. Unfortunately, at present, there are few packages and tools that capture both longitudinal data patterns as well as relationships between independent variables when constructing synthetic datasets.

From a practical standpoint, our educational primer also illustrated challenges and problems of embracing synthetic data generation of variables formulated as simple ratio statistics in sports and exercise sciences [50, 51]. While retained for illustrative purposes, the conceptual and statistical inconsistency of the acute-to-chronic workload ratio variable [25, 52] might have contributed to the introduction of undesired noise and errors in synthetic data generation processes that implicitly hindered and limited any replication of the original study outcomes [20]. The fact that alternative statistical approaches for clinical prediction model development could have been more suitable for exploring the association between training load and noncontact injury occurrence also deserves attention, although our educational primer attempted to explore the ability of synthetic data to replicate statistical outcomes from the models used in the original study [20].

## Conclusions

We provide the first primer exploring opportunities and challenges relevant to generating synthetic data to address questions in the context of injury and training load data, with this being an important first step toward better understanding of the utility of synthetic data for the sports and exercise sciences more broadly. Although synthetic data do not constitute a direct replacement for real data from conceptual and empirical standpoints, the value and information of synthetic data generation are contingent on researcher-based decisions and satisfying specific assumptions that are plausibly realistic and consistent with the context sought to be examined. In practice, the actionability of synthetic data generation is generally prone to whether the generation process of synthetic data was practically anchored to the statistical models used for specific types of analysis and exploration of synthetic data. In short, and for sport science and medicine, the following steps can be recommended:

1. A synthetic dataset’s goal should be clearly communicated. If the goal is to generate synthetic data generation that can be tested and analyzed for specific types of utility, then an appropriate generation process should be selected that maps to the specific utility of the dataset. If the goal is to maximize participant privacy while still making data open, consideration should be given to how synthetic data are generated and how they are released publicly.
2. Synthetic data should be accompanied by documentation of their generation process, including the predictors used, the model framework used for generation, and the potential limitations associated with this in terms of how the synthetic data could be explored as a part of future research;
3. As a community, we should develop appropriate processes for improving transparency around synthetic data generation for open release. Additionally, sport researchers wishing to generate fit-for-purpose synthetic datasets should partner with relevant expertise (from data science and statistics) to ensure that datasets made public can serve their intended purpose.
4. If a synthetic dataset is made available, researchers who are revisiting those data to make any claims must verify any outcomes of explorations of the real original dataset. In this sense, synthetic data should only be used to frame subsequent hypotheses that can be tested on the original data, rather than making these inferences using the synthetic data itself.

## Supplementary Information

Below is the link to the electronic supplementary material.

Supplementary file1 (DOCX 66 KB)

## Declarations

### Data Availability

No original data are publicly released with this article; however, as per Supplementary Material S5, synthetic datasets generated using two different sets of simulation conditions have been released with this manuscript and are available at: https://github.com/johnwarmenhoven/SynthData_in_Sport/tree/main/R-Code/Example%20Synthetic%20Datasets.
