# Supplementary material for: Synthetic Data for Sharing and Exploration in High-Performance Sport: Considerations for Application
Source: Sports Med. 2025 Jun 26;55(8):2019–37. doi: 10.1007/s40279-025-02221-6 (PMC12460485; doi:10.1007/s40279-025-02221-6)
Supplement: Supplementary file 1 — Supplementary file1 (DOCX 66 KB) [file 40279_2025_2221_MOESM1_ESM.docx]

**Supplement 1 (S1): Original data and research context (full description)**

***Purpose for using this dataset***

We have specifically revisited data from Fanchini et al. [20], which was also used in a subsequent study [28]. The data availability statement for this subsequent study stated, “*data cannot be shared since there is no permission from the team owning the data, but sharing of a synthetic dataset may be considered upon request*.” In the current study, we intended to create a synthetic dataset to provide enough information relevant to replicate the findings of the GEE model reported in both studies [20, 28]. As such, the present study provided a secondary data analysis of original training load and injury observations examined from the original study by Fanchini et al. [20].

***Original dataset***

From a general standpoint, the term “*load*” is typically referred to as the amount of physical training quantified in terms of running distances covered (at various speeds) during training and match, and/or other alternative surrogate determined using relevant psychometric measurement instruments [53, 54]. Specifically, Fanchini et al. [20] examined prospective data collected from a sample of 34 professional football players on the same team over 3 consecutive competitive seasons. Internal training load was assessed using session duration (minutes) and ratings of perceived exertion (RPE) using a level-anchored semi-ratio CR-10 Borg scale (Borg CR10®), with session duration x RPE (sRPE) as proxy measurement of interest (i.e., as a cumulative measure of exposure) [20, 55]. Weekly or acute training load was calculated as the most recent 7-day sRPEs period. Uncoupled chronic training loads were then calculated as 2-week, 3-week, and 4-week rolling averages. The original study of Fanchini et al. [20] also explored the relationship between training load and non-contact injury occurrence based on acute load (numerator) divided by the previous weeks’ chronic load periods (denominator) as a simple ratio index defined as the acute-to-chronic (uncoupled) workload ratio [20, 56]. All injuries during the data collection period were classified based on Fuller et al. [57]’s criteria, and defined as a binary outcome (yes injury=1, no injury=0). The present study retained the consideration of this simple ratio index for pure educational purposes even though there is lack of empirical support underpinning its calculation for research and applied purposes [25, 52]. As such, the synthetic dataset will contain five variables:

1. *WeekID*: A numeric variable specifying the week to which the observation belongs. WeekID ranges between 1 and 120 for each player. Each week must be a positive integer, i.e., the specific week in the testing period.
2. *PlayerID*: A dummy nominal variable outlining a specified player ID, ranging from 1 to 34.
3. *Acute Load*: a variable serving as a proxy for each player’s weekly training load.
4. *Chronic Load*: a variable serving as a proxy for each player’s 4-week chronic load for each week. Chronic load was uncoupled.
5. *Injury*: A binary variable indicating whether a player was injured during that week (yes=1, no =0).

***Original analysis***

Notwithstanding the different methods relevant to the analysis of repeated-measures data of predictor variables for clinical risk prediction [58-60], Fanchini et al. [20] explored the association between load and noncontact injury using a generalized estimating equations (GEE) approach. In the original work, the model included a logit link function and an exchangeable working correlation matrix based on lower quasi-likelihood under the independence model criterion [30]. This was attended to in all GEE analysis applied to synthetic data as part of the replication objectives.

**Supplement 2 (S2): Synthetic Data Generation Process using Synthpop**

When replicating a dataset with Synthpop, we assume the observed data is a random sample from a population. We can estimate the population's parameters using the sample, and then simulate a synthetic dataset based on those parameters. The process of simulating a synthetic dataset using synthpop depends on first defining distributions, where the joint distribution being synthesised is defined using a series of conditional distributions. First, a set of columns (i.e. variables) that are not to be synthesised are specified, with these referred to a *z_obs_*. These form the initial set of possible variables that can be used as predictors in defining conditional distributions for variables being synthetically generated but will not be generated synthetically themselves. *x_obs_* are *ALL* predictors used in the estimation of a conditional distribution for a given synthetic variable (therefore initially *z_obs_* = *x_obs_*). The process of generating synthetic data follows a sequential variable-by-variable process. In each instance the variable being estimated is referred to as *y_obs_*. For the first variable *y_obs_1_*, the selected *x_obs_* columns are used to predict the distribution of *y_obs_1_*. Once the distribution of *y_obs_1_* is predicted, it is added to the set of *x_obs_*. Then the next variable is selected, and its distribution is estimated conditional on the current set of *x_obs_ (which now includes* the estimated *y_obs_1_)*. The distribution of subsequent variables (subsequent *y_obs_n_*) follows this pattern, with every future variable being estimated conditional on initial *x_obs_* and all previous columns of predicted *y_obs_*. As such, variables within the synthetic datasets are predicted sequentially as they appear in the original dataset unless otherwise specified. For a comprehensive description of the Synthpop software please see these references [14, 34, 61].

Within synthpop, it is possible to specify different model generation frameworks for each variable, including parametric (e.g., linear regression) and non-parametric approaches (e.g., classification and regression trees (CART), random forest models, etc). A full outline of the possible model generation frameworks can be found in synthpop’s documentation [61]. In the present study, only CART was explored for generating each variable in each dataset, as this was the simplest non-parametric method and synthpop’s default. Within synthpop, the variable synthesis order is determined by the visit.sequence parameter and the synthesising model’s predictors are determined by the predictor.matrix parameter. Importantly, the visit sequence limits predictor variable choices since the model cannot condition on variables that have not yet been generated. Therefore, one should introduce important predictors early in the sequential process so they can be used to define the conditional distributions of future, less informative variables.

**Supplement 3 (S3): Metrics for exploring synthetic data**

***What is utility?***

There are two broad ways to assess the quality, or utility, of synthetic data. Firstly “*global utility*” assesses the overall similarity of the distributions of the specified variables within the synthetic data to the original data, independent of any specific research question. Alternatively, “*specific utility*” assesses the ability of the synthetic data to answer a specific research question or replicate the results of a specific analysis.

***Global utility***

Three common global utility metrics use a prediction model (the default in synthpop being a logistic regression, which was used in the present study) to determine whether data originated from the source dataset or the synthetic dataset. These metrics employ the use of a propensity score [31, 32], which represents the probability of each observation being real or synthetic. “*High*” global utility implies that the datasets are indistinguishable. These three metrics are as follows:

*pMSE*: A propensity score is estimated for each of the rows of the combined data, as the probability of classification for an indicator variable denoting whether the observation belonged to the real or synthetic dataset. The mean-squared difference between these estimated probabilities and the true proportion of records from the synthetic data in the combined data (denoted by *c*; usually 0.5) gives the utility statistic (1/*N*)Σ(*p*_i_ – *c*)^2^, which can then be used to calculate the propensity score mean-squared error *p*MSE [31]. A value close to 0 indicates strong global utility.

*s-pMSE*: the standardized pMSE is calculated to assess the distributional similarity between the distribution of the observed data and the model used to generate the synthetic data. It is calculated as the difference of the *p*MSE from its null expectation in units of the estimated null standard deviation. The *s-p*MSE an expectation of 0 and a standard deviation of 1 in the null case, with increased values of these statistics being expected if correct synthesis does not hold [31].

*PO50*: is the percentage of the observations correctly predicted over 50%, where the majority of observations in each grouping are in agreement with the original category (real or synthetic) of the observation [33]. Lower *PO50*s have a better global utility. In other words, the statistical model cannot reliably discern original observations from synthetic ones.

***Specific utility***

Specific utility refers to measures used to evaluate whether analyses using synthetic data replicate a specific analysis performed on the original data. In the present study specific utility was looked at in terms of replication of GEE outcomes from the original work of Impellizzeri et al. [28]. For the replication of GEE outcomes, three metrics were evaluated.

*MAE of GEE estimate*: MAE was calculated comparing the Acute-Chronic (4-week) training load ratio GEE parameter estimate in the synthetic and original datasets, mirroring the analysis in Fanchini et al. [20] for creating this metric.

*MAE of GEE SE*: MAE was calculated comparing the Acute-Chronic (4-week) training load ratio GEE parameter’s standard error (SE) in the synthetic and original datasets, mirroring the analysis applied in Fanchini et al [20].

*MAE of GEE p-values*: MAE was calculated as the average difference between *p*-values from the GEE fitted on the original data, and equivalent p-values fitted on the synthetic data.

For assessing how well temporal structures were preserved in the synthetic workload data, specific error metrics were calculated for load variables.

***Additional metrics***

*Computation time*: Although not a metric used to assess the quality of the synthetic data, it was included to assess the feasibility for using a given set of specifications for generating synthetic data. Computation time is the time taken to run the synthetic data generation script for a single dataset.

*MAE of acute load and chronic load observations*: Given that the data were panel (i.e., a large number of repeated measures for each individual across weeks), mean absolute error (MAE) measures were calculated as the distance between the original and synthetic datasets for acute load and chronic load (across all observations). This was only conducted in the first 4 simulation conditions, given that injury remained fixed and did not have new synthetic data generated. As such, MAE served as a general measure of fit to evaluate how much Acute Load and Chronic Load variables varied at the observation level, across a range of varying experimental specifications in the first four simulation conditions. Larger MAE indicate that the temporal structure within each individual was not well preserved and possessed less utility from the perspective of preserving temporal structures.

**Supplement 4 (S4): Exploring the variability of the data generation processes**

For simulation conditions one to four, the variability of the underlying data generation process within each simulation condition was compared across all synthetic datasets generated. This involved comparing synthetic datasets as a series of adjacent pairs (i.e. dataset 1 and dataset 2, dataset 2 and dataset 3, and so on). For each set of adjacent pairs, the same metrics were calculated for each pair, providing an indication of whether a simulation condition was likely to be more inconsistent, regardless of its performance for specific utility when assessed relative to the original data.

The results for this series of tests are presented alongside the results of specific utility for the same four simulation conditions, with both being presented below in Table S1 (original specific utility and additional metrics results relative to the original data), and Table S2 (specific utility and additional metrics relative to the error between pairs of synthetic datasets).

| **Table S1**. Original specific utility and additional metric MAEs compared to original data. | | | | | |
| --- | --- | --- | --- | --- | --- |
|  |  | **Synthetic Training Load Data Simulations** | | | |
|  |  |  |  |  |  |
|  |  | **Base (1)** | **Base_Week (2)** | **Time_Lag_1wk (3)** | **Time_Lag_3wks (4)** |
|  |  | *MAE (SD)* | *MAE (SD)* | *MAE SD)* | *MAE (SD)* |
| **Specific Utility (MAE)** | | | | | |
| **Chronic load simulated as "independent variable"** | **GEE Estimate** | 0.37 (0.27) | 0.37 (0.26) | 0.48 (0.29) | 0.75 (0.32) |
|  | **GEE SE** | 0.1 (0.06) | 0.11 (0.07) | 0.11 (0.07) | 0.14 (0.07) |
|  | **p-value** | 0.03 (0.07) | 0.12 (0.20) | 0.36 (0.28) | 0.57 (0.27) |
| **Chronic load calculated from synthetic acute load** | **GEE Estimate** | 0.33 (0.27) | 0.75 (0.40) | 0.83 (0.35) | 1.16 (0.34) |
|  | **GEE SE** | 0.13 (0.08) | 0.13 (0.07) | 0.12 (0.07) | 0.1 (0.06) |
|  | **p-value** | 0.11 (0.17) | 0.48 (0.28) | 0.55 (0.27) | 0.49 (0.29) |
| **Acute load** | **Observation level** | 462.57 (6.82) | 365.65 (6.40) | 330.59 (6.10) | 295.02 (5.62) |
| **Chronic load simulated** | **Observation level** | 272.26 (4.42) | 208.28 (3.82) | 142.12 (2.63) | 126.03 (2.50) |
| **Chronic load calculated** | **Observation level** | 257.05 (5.77) | 196.81 (4.49) | 194.92 (4.35) | 180.86 (4.19) |
|  |  |  |  |  |  |
|  |  |  |  |  |  |
| **Table S2.** Original specific utility and additional metric MAEs compared to between pairs of synthetic data. | | | | | |
|  |  | **Synthetic Training Load Data Simulations** | | | |
|  |  |  |  |  |  |
|  |  | **Base (1)** | **Base_Week (2)** | **Time_Lag_1wk (3)** | **Time_Lag_3wks (4)** |
|  |  | *MAE (SD)* | *MAE (SD)* | *MAE SD)* | *MAE (SD)* |
| **Specific Utility (MAE)** | | | | | |
| **Chronic load simulated as "independent variable"** | **GEE Estimate** | 0.40 (0.32) | 0.50 (0.35) | 0.34 (0.28) | 0.37 (0.29) |
|  | **GEE SE** | 0.09 (0.06) | 0.09 (0.06) | 0.08 (0.06) | 0.09 (0.07) |
|  | **p-value** | 0.05 (0.09) | 0.17 (0.22) | 0.30 (0.24) | 0.29 (0.22) |
| **Chronic load calculated from synthetic acute load** | **GEE Estimate** | 0.50 (0.37) | 0.46 (0.35) | 0.39 (0.28) | 0.38 (0.28) |
|  | **GEE SE** | 0.1 (0.08) | 0.09 (0.07) | 0.08 (0.06) | 0.08 (0.06) |
|  | **p-value** | 0.15 (0.19) | 0.32 (0.24) | 0.30 (0.22) | 0.34 (0.23) |
| **Acute load** | **Observation level** | 462.58 (8.07) | 366.4 (7.49) | 331.49 (6.57) | 295.2 (5.93) |
| **Chronic load simulated** | **Observation level** | 273.04 (4.70) | 222.17 (4.49) | 142.94 (3.04) | 129.08 (2.96) |
| **Chronic load calculated** | **Observation level** | 257.06 (5.78) | 196.8 (4.49) | 194.91 (4.35) | 180.87 (4.19) |

Even though specific utility was best in simulation condition one (when calculating chronic load from acute synthetic load) for error in the GEE estimate when compared to the original data, the variability of the GEE estimate error when calculated relative to adjacent synthetic dataset pairs was the worst (i.e. highest) for simulation condition one. This trend was reversed for simulation condition four, with the GEE estimate error being the highest when calculated relative to the original data, but the lowest when calculating the error relative to adjacent pairs of synthetic data.

What this indicates is that despite having a synthetic data generation process, which provides better specific utility, like simulation condition one, there is still a possibility that the data generation process may be highly variable and inconsistent from synthetic dataset to synthetic dataset, despite centring around an average error that indicates good specific utility.

Despite this occurring for specific utility in simulation condition one, the trend for acute load error and chronic load errors to descend in magnitude across the simulation conditions (being highest in simulation condition one) held consistently when calculating error relative to adjacent synthetic dataset pairs. This indicated that the variability of the synthetic data generation processes decreased as more temporal predictors were added into data generation processes.

**Supplement 5 (S5): Example of synthetic datasets being generated and released**

Synthetic datasets for two simulation conditions were generated from the original dataset used in the present study and released openly. These are available for use at: <https://github.com/johnwarmenhoven/SynthData_in_Sport/tree/main/R-Code/Example%20Synthetic%20Datasets>.

For these two simulation conditions, there are different specifications for the generation of synthetic data. Each set contains 10 datasets. This number of datasets per simulation conditions was selected to allow for repeated testing across a number of datasets within a condition, while being a small enough number that it mitigated against the risk of identifying the original observations by pooling the synthetic datasets together. Details of these datasets are below.

**Table S1**. Simulation condition specifications for the two sets of synthetic data that has been released on GitHub. Table follows a similar layout to Table 1 in the main manuscript.

|  | **Variables for Xobs** | **Variables for Yobs** | **Visit Sequence of Predictors** | **Description & Rationale** |
| --- | --- | --- | --- | --- |
| **Simulation Condition A** | Injury; PlayerID; Acute load; Chronic load; Injury. | Acute load; Chronic load; Injury | Injury; PlayerID; Acute load; Chronic load. | This is the same as simulation condition one in the main manuscript, but with the exception that this also simulates injuries (not just workload variables).  **Rationale**: This is a simple set of specifications for synthetic data generation, designed to provide a dataset that reports similar outcomes to the GEE model applied to the original data. So if for example a new researcher wanted to verify the analysis and code on this synthetic data for the original GEE model, or make a minor modification to the GEE model (e.g. a different link function), these datasets would be appropriate for this. |
| **Simulation Condition B** | Injury; PlayerID; WeekID; Acute load; Chronic load; Injury | Acute load; Chronic load; Injury | AL_Lag(1-step); AL_Lag(2-step); AL_Lag(3-step); CL_Lag(1-step); CL_Lag(2-step); CL_Lag(3-step); Acute load; Chronic load; Injury. | This is the same as simulation condition seven in the main manuscript. PlayerID was removed from the visit sequence and injury was used as the final variable to be synthetically generated.  **Rationale**: this simulation condition allows researchers an ability to look at any autoregressive features leading into injuries, given that autoregressive variables were included in the generation process. These 10 datasets are however unlikely to provide outcomes for the GEE that are consistent with the original dataset. |

For any intentions using these synthetic datasets, outside of the applications specified in the table above, the results are likely to be erroneous. Additionally, even though details on these specifications are provided above, and guidance in what these datasets can be used for is also provided, there is still likely to be inconsistencies in the outcomes of models applied to these datasets, given the large amount of variability demonstrated in the data generation processes highlighted in Supplement 4.

As such, all findings on these datasets must be verified on the original data, under the direction and guidance of the original custodians of the data (being the research team on the current project).
